# Supplementary material for: Plasmon mediated coherent population oscillations in molecular aggregates
Source: Nat Commun. 2023 Dec 5;14:8035. doi: 10.1038/s41467-023-43578-4 (PMC10698180; doi:10.1038/s41467-023-43578-4)
Supplement: Supplementary file 1 — Supplementary Information [file 41467_2023_43578_MOESM1_ESM.pdf]

Supplementary Information for

Plasmon mediated coherent population oscillations in molecular aggregates

Daniel Timmer[1+], Moritz Gittinger[1+], Thomas Quenzel[1], Sven Stephan[1,6], Yu Zhang[2], Marvin F. Schumacher[3], Arne Lützen[3], Martin Silies[1,6], Sergei Tretiak[2], Jin-Hui Zhong[1,7], Antonietta De Sio[1,4] and Christoph Lienau*[1,4,5]

[1]Institut für Physik, Carl von Ossietzky Universität, Oldenburg, Germany.

[2]Theoretical Division, Los Alamos National Laboratory, Los Alamos, NM, USA.

[3]Kekulé-Institute for Organic Chemistry and Biochemistry, University of Bonn, Bonn, Germany.

[4]Center for Nanoscale Dynamics (CeNaD), Carl von Ossietzky Universität, Oldenburg, Germany.

[5]Forschungszentrum Neurosensorik, Carl von Ossietzky Universität, Oldenburg, Germany.

[6]Present Address: Institute for Lasers and Optics, University of Applied Sciences, Emden, Germany.

[7]Present Address: Department of Materials Science and Engineering, Southern University of Science and Technology, Shenzhen, Guangdong, China.

[+]These authors contribute equally to this work.

Correspondence to: christoph.lienau@uni-oldenburg.de

Table of Contents

Supplementary Note 1: ProSQ-C16 squaraine based J-aggregates 2
Supplementary Note 2: Preparation of nanoslit arrays covered with a J-aggregated squaraine thin film 2
Supplementary Note 3: Angle-resolved linear reflectivity of gold nanoslit arrays 3
3.1. Measurements 3
3.2. FDTD simulations 3
Supplementary Note 4: Experimental Methods 4
Supplementary Note 5: Additional 2DES data for different angles 5
Supplementary Note 6: Pump-probe maps recorded for different incidence angles 7
Supplementary Note 7: Spatial field distributions and linear dispersion relations 7
7.1. Spatially homogeneous thin films 7
7.2. Nanoslit arrays 9
7.3. Anisotropic exciton-plasmon coupling 11
7.4. Strongly and weakly coupled excitons 12
Supplementary Note 8: Frenkel exciton simulations 13

8.1. Reduced Hamiltonian 14
8.2. Simulating the optical spectra and dynamics upon optical excitation 14
8.3. Simulation parameters 15
Supplementary Note 9: Simulation of 2DES and pump-probe spectra 16
9.1. Plasmon 17
9.2. Excitons 18
9.4. Total Hamiltonian 20
9.5. Simulation parameters 21
9.6. Additional simulation results 21
Supplementary Note 10: UP-LP Rabi oscillations 22

Supplementary Note 1: ProSQ-C16 squaraine based J-aggregates

(S,S)-enantiomers of 2,4-Bis[4-((S))-2-(hexadecyloxymethyl)-pyrrolidone-2,6-dihydroxyphenyl]squaraine molecules (ProSQ-C16, Fig. 1b, inset) have been synthesized as described by Schulz et al. [1].

These squaraine molecules are quadrupolar D−π−A−π−D charge transfer dyes consisting of a center squaric acid connected to two aniline donor arms [2]. In solution, the optical absorption spectrum shows mainly a single electronic transition in the visible with a large molar extinction of ~300.000 $Lmol^{-1}cm^{-1}$ at ~1.9 eV with very weak vibronic substructure. Other electronically excited states are energetically well separated, in agreement with predictions by essential state models [3, 4]. The strong concentration in oscillator strength, weak vibronic coupling and sparsity of electronic states makes these molecules interesting model systems for J-aggregated thin films with spectrally sharp optical resonances [3].

After spin-coating and thermal annealing, thin films of ProSQ-C16 display a very pronounced, red-shifted J-aggregate band at ~1.6 eV in their linear optical absorption (Fig. 1b). For optimized annealing conditions, this band displays a large circular dichroism [2, 3]. The linear optical properties of the J-aggregated films are reasonably well described within a Frenkel-exciton model [5]. This model assumes a linear chain of two-level-systems, coupled by dipolar nearest-neighbor interactions that delocalize the excitonic excitations across the chain [5]. Site disorder results in Anderson localization of J-aggregate excitons and thus a broadening of the linear absorption. When using gold instead of glass as a substrate for the aggregated film, exciton coupling to the surface plasmon polaritons (SPP) of the planar gold film further delocalizes the exciton wavefunction and dramatically reduces the linewidth of the optical resonance [6]. Such a disordered Frenkel exciton model has been successfully used to explain two-dimensional electronic spectroscopy (2DES) maps of ProSQ-C16 thin films [5].

Supplementary Note 2: Preparation of nanoslit arrays covered with a J-aggregated squaraine thin film

A planar, polycrystalline gold film with a thickness of ~200 nm has been deposited on a fused silica substrate by electron beam evaporation. Thermal annealing has been used to enhance the grain size [5, 7, 8]. Nanoslit arrays with a grating period of $a_0 = 530\ \mathrm{nm}$ were fabricated by focused gallium ion beam milling (Helios NanoLab 600i, FEI). The slits have depths of 45 nm and widths of 45 nm. The area size is 80 x 150 μm^2. The structured gold film was covered with a ~10-nm-thick J-aggregated thin film, prepared by spin-

coating of a solution of ProSQ-C16 squaraine molecules in chloroform onto the sample. The sample was subsequently annealed at a temperature of 120°C for 90 minutes, following the recipe reported in [5]. To perform reference optical measurements on the bare gold substrate, a part of the squaraine film was removed using chloroform.

Supplementary Note 3: Angle-resolved linear reflectivity of gold nanoslit arrays

3.1. Measurements

To characterize the manufactured gold nanoslit arrays, we recorded angle-resolved linear reflectivity spectra before covering the sample with the J-aggregate layer. For this, we used a supercontinuum white light source (SC400-4, Fianium) that was focused onto the structured area under an angle of incidence θ (see Fig. 1a of the main manuscript). To allow for surface-plasmon polariton (SPP) excitation, the polarization was set to be perpendicular to the slit orientation (p-polarization). The reflected beam was then collected and measured using a fiber spectrometer. Fig. S1a presents the measured linear reflectivity for different θ, using the bare gold film as a reference. We observe a distinct and narrow SPP resonance with a linewidth of 16 meV at $\theta = 21°$. This resonance results from the coupling of the incident light with in-plane momentum $k_{x,L} = (\omega/c_0)\sin\theta$ to the AM[-1] SPP mode at the air-metal interface with in-plane momentum $k_x = k_{x,L} - 2\pi/a_0$ [5, 9]. Both SPP energy and linewidth decreases with increasing angle of incidence. The resonance energy follows the AM[-1] branch of the SPP dispersion relation. The reduction in linewidth reflects the reduced radiative SPP damping with decreasing SPP energy [10].

The angle-dependent linear reflectivity spectra of the nanoslit sample coated with a squaraine thin film that are shown in Fig. 1e of the main manuscript have been measured using the same approach.

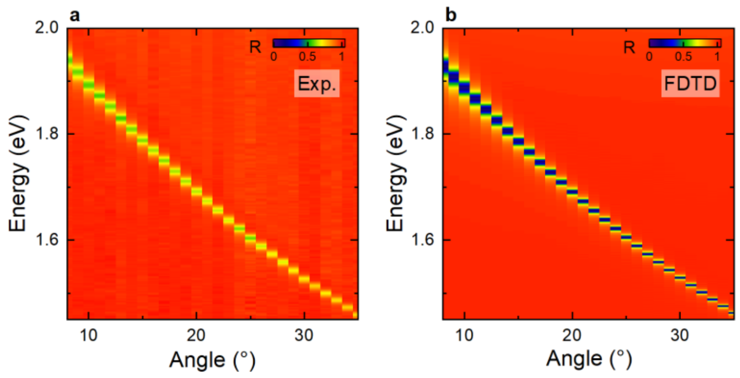

Supplementary Figure 1: *Angle-resolved linear reflectivity spectra of a nanoslit array with slit depth and width of 45 nm and a grating period of $a_0 = 530\ nm$. a) Measurement and b) FDTD simulation.*

3.2. FDTD simulations

We performed Finite-Difference Time-Domain (FDTD) simulations of the angle-dependent linear reflectivity spectra of gold nanoslit arrays using the commercial Maxwell equation solver Lumerical FDTD Solutions. The geometrical parameters of the nanoslit arrays were the same as in the experiment. Due to the translational invariance along the slits, it is sufficient to perform two-dimensional simulations of the system. To retrieve linear reflectivity spectra, a broadband plane-wave is injected into the simulation box, impinging onto the sample surface under a fixed angle θ. A linear array of monitors is placed above the injection-plane of the incident plane wave to record the reflected fields. Normalization to the incident field gives the reflectivity of the sample. Field monitors are used to map the local electric field in the plane of

incidence. Periodic boundary conditions are applied to the lateral boundaries, and perfectly matched layers are used at the top and bottom boundaries. To ensure incidence angles that are independent of the photon energy, the simulation is performed using the Broadband Fixed Angle Source Technique (BFAST) [11]. In the simulations, the dielectric function of gold is taken from [12]. The resulting angle-dependent linear reflectivity spectra (Fig. S1b) are in reasonable agreement with the experimental ones.

The same simulations have been carried out for a nanoslit array covered with a 10-nm thin film of J-aggregate ProSQ-C16 squaraine molecules. The dielectric function of the J-aggregated thin film is taken from [13]. Again, a good agreement between simulation (Fig. 1f) and experiment (Fig. 1e) can be observed.

Supplementary Note 4: Experimental Methods

Pump-probe (PP) and 2DES measurements were performed using a home-built setup previously described in [2]. To allow for rapid data acquisition, the laser system, a Tangerine V2 (Amplitude Systèmes), is operated at a repetition rate of 175 kHz. It delivers 200-µJ-pulses at 1030 nm with a duration of 260 fs (full width at half maximum of the intensity profile). These pulses pump a home-built Noncollinear Optical Parametric Amplifier (NOPA) [5, 14], creating broadband, 0.5-µJ-pulses with a spectrum spanning from 650 nm to 900 nm (Fig. S2a). Pulse characterization by second harmonic frequency resolved optical gating (SH-FROG) gives a pulse duration of ~12 fs (Fig. S2b).

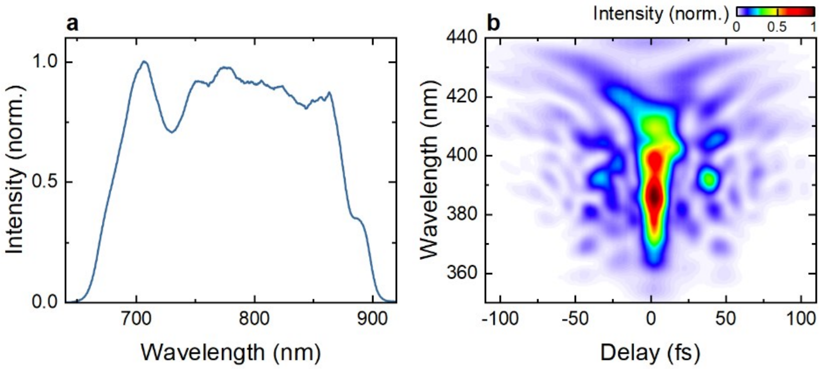

Supplementary Figure 2: *a) Experimental NOPA spectrum. b) SH-FROG map, measuring the cross-correlation between the pump and probe pulse at the sample position, yielding a retrieved pulse duration of ~12 fs.*

We use these NOPA pulses as both the pump and the probe in a home-built 2DES setup. A phase-stable and collinear pump-pulse pair with variable delay τ (coherence time) is generated by an interferometer based on birefringent wedges (TWINS, Translating Wedge-based Identical pulse eNcoding System [5]). The pump pulses are periodically switched on and off at 43.75 kHz by a mechanical chopper system (MC2000B, Thorlabs) equipped with a custom-made blade (500 slots). The vertically aligned and p-polarized pump and probe beams are focused onto the sample to a spot size of ~60x60 µm² under the same angle of incidence θ and with a small angle mismatch in the orthogonal direction (see Fig. 1a). The angle of incidence θ of pump and probe pulses is tuned in synchrony using a rotating sample mount. The reflected probe beam is then collected and sent to a monochromator (Acton SP2150i, Princeton Instruments) with an attached fast line camera (Aviiva EM4, e2v) allowing to record probe spectra S at an acquisition rate of 87.5 kHz (i.e., at half the laser repetition rate) as a function of the detection energy E_{det}. We thus record a differential reflectivity spectrum $\Delta R/R$ from a set of 4 laser shots by taking the difference between spectra with (S_{on}) and without (S_{off}) pump

$$\frac{\Delta R}{R}(\tau, T, E_{det}) = \frac{S_{on}(\tau, T, E_{det}) - S_{off}(E_{det})}{S_{off}(E_{det})}. \quad (1)$$

Here, the waiting time T denotes the delay between the second pump and the probe pulse. This delay is controlled using a motorized linear translation stage (M126.DG1, Physik Instrumente). For the 2DES measurements, at each waiting time T, the coherence time is scanned and differential spectra are recorded on the fly, whereas for the pump-probe experiments, we fix the coherence time to $\tau = 0$ fs and scan only the waiting time T.

To calculate absorptive 2DES maps [15]

$$A_{2D}(E_{ex}, T, E_{det}) = \Re\left(\int_{-\infty}^{\infty} \Theta(\tau) \frac{\Delta R}{R}(\tau, T, E_{det}) \mathrm{e}^{iE_{ex}\tau/\hbar} \mathrm{d}\tau\right) \quad (2)$$

a Fourier transform of the differential reflectivity is performed along the coherence time to obtain the 2DES spectra as a function of the excitation energy axis E_{ex}. Here, $\hbar$ denotes Planck's reduced constant and the Heaviside function $\Theta(\tau)$ ensures that only data recorded for positive coherence times contribute to the 2DES spectrum.

To characterize the time resolution of the experiment, we have placed a 10 µm thick beta barium borate crystal at the sample position and recorded the cross-correlation SH-FROG map shown in Fig. S2b. Experiments of the squaraine J-aggregate on gold and on the gold nanoslit arrays are performed at room temperature in air using pump and probe fluences of 10 µJ/cm². We have carefully checked, by varying the pulse fluences, that all measurements are performed within the regime of a third-order nonlinear optical polarization [16].

Supplementary Note 5: Additional 2DES data for different angles

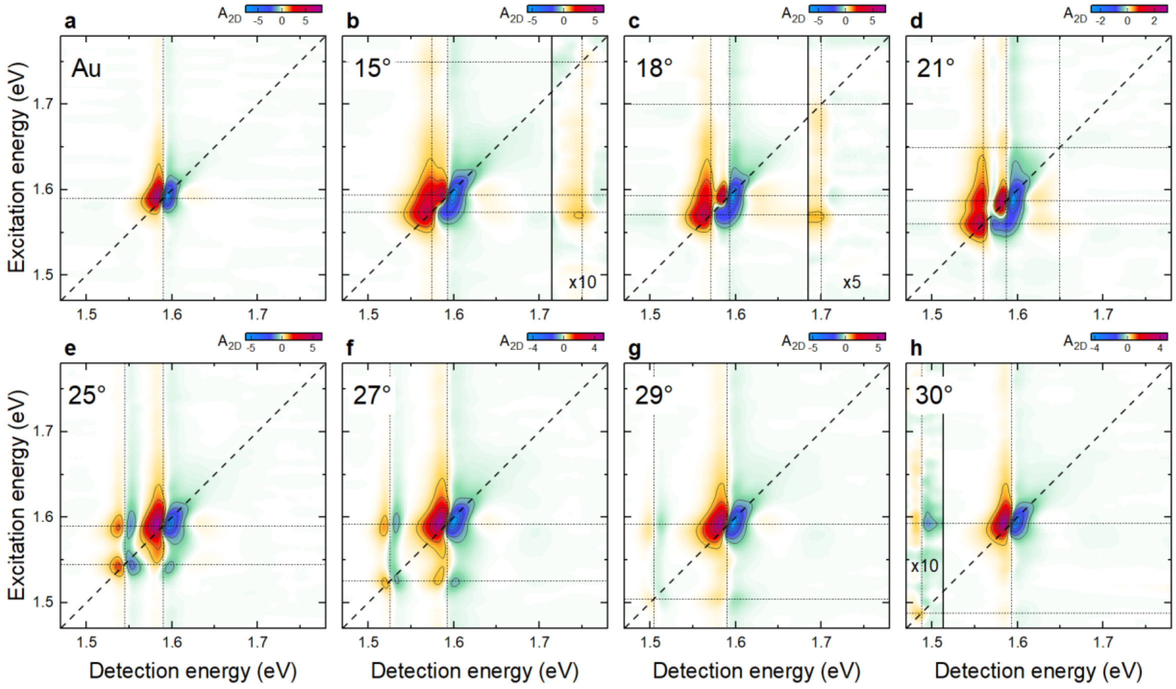

Supplementary Figure 3: *Experimental 2DES maps at T = 0 fs for squaraine J-aggregate on a bare gold film (a) and on the gold nanoslit array (b-h), recorded for different angles of incidence. The energetic positions of the upper (UP) and lower (LP) polariton states are marked, together with the "uncoupled" exciton (X), as dotted lines. The measurements highlight the variation of the*

polariton-related diagonal and cross peaks with angle of incidence. The "uncoupled X" peak at $E_X \approx 1.59\ eV$ is virtually independent of angle.

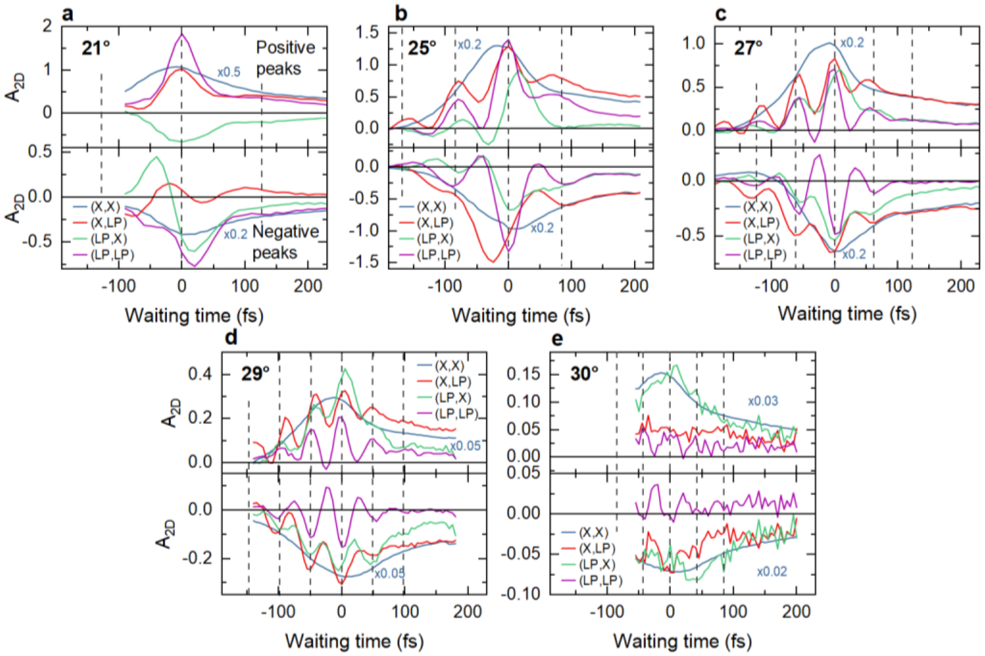

Supplementary Figure 4: *Waiting time dynamics extracted from angle-resolved 2DES maps recorded for a squaraine J-aggregate film on the gold nanoslit array. Peak dynamics for positive and negative diagonal and cross peaks of X and LP for angles of 21° (a), 25° (b), 27° (c), 29° (d) and 30° (e). For all angles, pronounced temporal oscillations of the 2DES signal occur with a period of T_X, given by the splitting between "uncoupled" exciton and lower polariton states. Oscillations at the Rabi period T_R are much weaker in amplitude. The oscillation periods are marked with dashed lines. The amplitude of the (X,X) dynamics has been scaled to match that of the remaining traces. The LP-associated 2DES peaks show a strong reduction in amplitude with increasing angle of incidence.*

Supplementary Note 6: Pump-probe maps recorded for different incidence angles

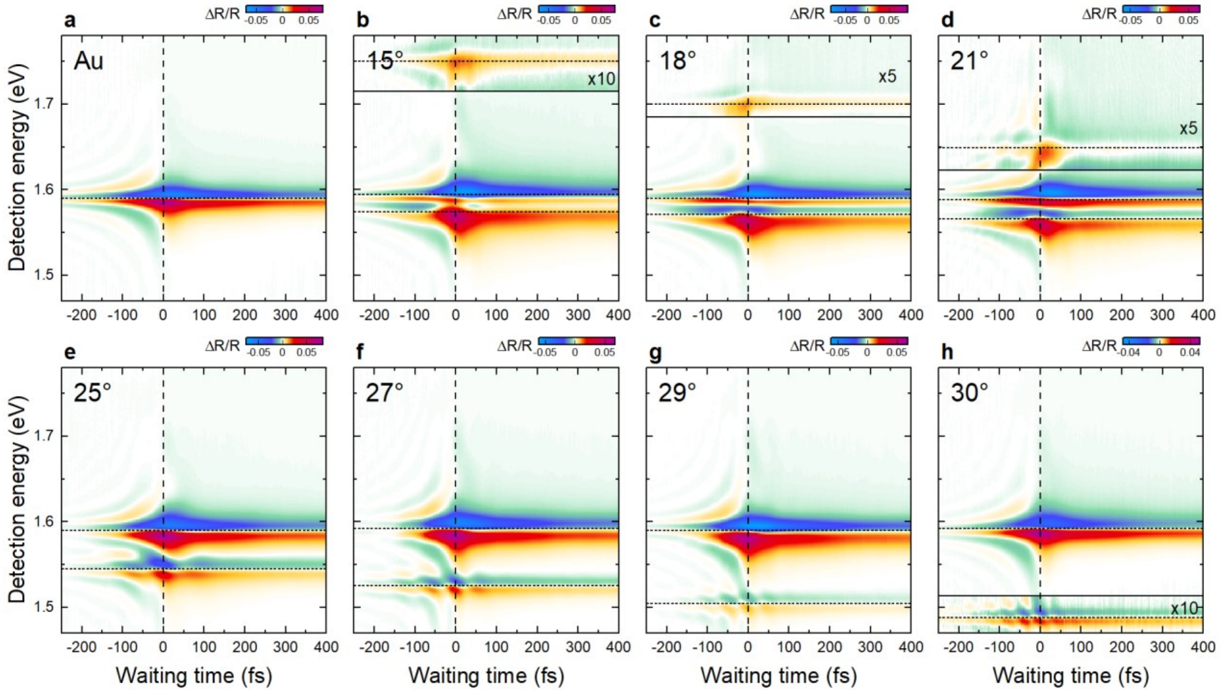

Supplementary Figure 5: *Experimental pump-probe maps for squaraine J-aggregates on a bare gold film (a) and on the gold nanoslit array (b-h) recorded for different angles of incidence. While the dispersive LP feature can be observed for all angles, the weak UP signal is only seen for angles between 15°, and 21°, below the crossing angle of 23°.*

Supplementary Note 7: Spatial field distributions and linear dispersion relations

A more quantitative analysis of the local optical near fields of the gold nanoslit array is essential to understand how the dipolar coupling to the SPP affects excitons at different positions on the sample surface. To this aim, we simulated the electric fields of different hybrid samples using Lumerical FDTD Solutions.

In these two-dimensional simulations, the x-direction lies in the sample plane, perpendicular to the slit axis, and the z-direction is normal to the sample plane. The samples are illuminated using the BFAST technique under an angle of incidence of 23°. The local field distributions are investigated for selected wavelengths.

7.1. Spatially homogeneous thin films

We start by analyzing the field distribution of a planar gold film in air without coating. The local fields at a wavelength of 783 nm, close to the J-aggregate resonance, are depicted in Fig. S6 (left). We then add a 12-nm-thin coating layer with a spectrally constant, real-valued dielectric function of $\varepsilon_{Diel} = 4$ (Fig. S6 right). This simulation tests the effect of an off-resonant coating on the field distribution. In Fig. S7, the dielectric function is replaced by $\varepsilon_{Jagg}(\omega) = 1 + \chi_{Jagg}(\omega)$, the dielectric function of the J-aggregate layer taken from [5].

The boundary conditions at the interface between gold and a generic dielectric material relate the electric field ($\mathbf{E}$) components of both sides of the interface via [2]

$$\mathbf{n} \times (\mathbf{E_{Au}} - \mathbf{E_{Diel}}) = 0 \quad (3)$$
$$\mathbf{n} \cdot (\mathbf{D_{Au}} - \mathbf{D_{Diel}}) = \sigma, \quad (4)$$

where $\mathbf{n}$ is the normal vector of the interface between the two layers with dielectric functions ε_{Au} and ε_{Diel}. The electric displacement field is $\mathbf{D} = \varepsilon\varepsilon_0\mathbf{E}$ and σ is the surface charge density, which we assume to be zero. The in-plane electric field components in the two layers are therefore identical at the interface, while their normal components are related via the ratio of the dielectric functions. This reduces the normal fields, in z-direction, inside the dielectric layer in Fig. S6 (right) by a factor of 4, as can be seen in the crosscuts in panels b and c (red). When changing the dielectric layer to a J-aggregated squaraine film (Fig. S7), the reduction in z-component of the field is even more pronounced due to the large on-resonance value of the dielectric function of the J-aggregated squaraine film [17].

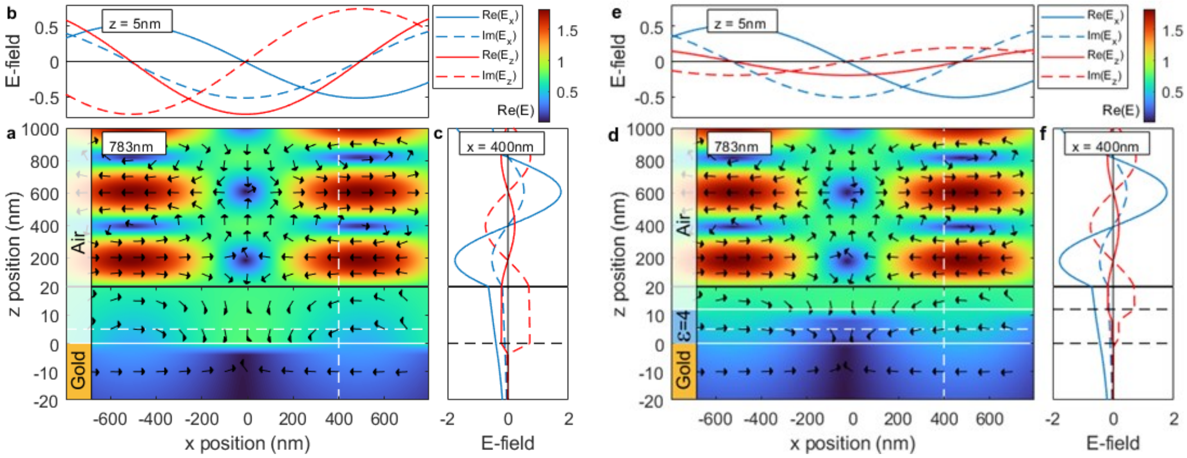

Supplementary Figure 6: *a),b),c) Electric field distribution across the interface between a planar gold film and air. d),e),f) Gold film covered with a 12-nm-thick coating with $\varepsilon_{Diel} = 4$. a) Real part of the local field at 783 nm, close to the J-aggregate resonance. The color map represents the magnitude of the field, while the arrows depict the field orientation. b) Crosscuts at a constant height of $z = 5\ nm$ above the gold interface, depicting the real (constant line) and imaginary (dashed line) parts of the x (blue) and z (red) components of the field. c) Same as in b) but for a vertical crosscut at $x = 400\ nm$. Panels d), e) and f) present the same as a), b) and c) but for the coated gold film.*

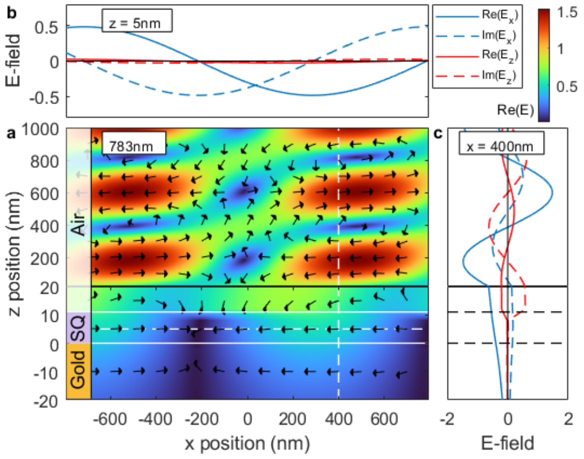

Supplementary Figure 7: *Same as in Fig. S6 (right) for a gold film covered by a 10-nm thick J-aggregated squaraine film with a dielectric function taken from [2]. a) Real-part of the electric field at 783 nm. b) Crosscuts at $z = 5\ nm$. c) Crosscuts at $x = 400\ nm$. At the squaraine exciton resonance (783 nm), the reduction of the z-component is more pronounced than in Fig. S6 (right).*

The metallic properties of the gold dielectric function allow for SPP excitations at planar gold-air or gold-dielectric interfaces. The dispersion relation of these SPPs [2]

$$k_x^2 = \frac{\varepsilon_{Au}\varepsilon_{Diel}}{\varepsilon_{Au}+\varepsilon_{Diel}}\frac{\omega^2}{c^2}, \quad (5)$$

with in-plane wavevector k_x, angular frequency ω and speed of light c, lies outside the light line. Thus, the SPPs of a planar interface cannot be excited by far-field plane wave radiation and, therefore they are not seen in Figs. S6 and S7. Consequently, plasmon-induced field enhancement is absent in Figs. S6 and S7 and the field amplitude is of the same order of magnitude as that of the incident far-field light.

7.2. Nanoslit arrays

To bridge the momentum mismatch between far-fields and evanescent SPP fields, we take advantage of a subwavelength periodic structure, a nanoslit array. This array provides phase-matching between the incoming plane wave fields and those of the SPP for a specific photon energy and angle of incidence. A simulation of the field distribution for such a structured gold film in contact with air is depicted in Fig. S8 (left). At a wavelength of 757 nm, in resonance with the SPP at 23° angle of incidence, the excitation of a delocalized plane wave SPP mode can be observed. The SPP electric field inside the dielectric (z > 0) at a planar interface

$$\mathbf{E_{SPP}} \propto \mathrm{e}^{-\kappa_z z}\left(\mathbf{n_x} + i\frac{k_x}{\kappa_z}\mathbf{n_z}\right)\mathrm{e}^{ik_x x - i\omega t}, \mathrm{z} > 0 \quad (6)$$

propagates in x-direction with k_x and decays exponentially in z-direction with

$$\kappa_z = \sqrt{k_x^2 - \varepsilon_{Diel}\frac{\omega^2}{c^2}}, \quad (7)$$

on the order of 580 nm in the depicted case [17]. An important effect of the excitation of SPPs is a strong field enhancement close to the gold surface on the order of 10, as seen by comparing Figs. S6 and S8. This reflects the excitation of the $\mathrm{AM}[-1]$ mode of the grating with momentum $k_x = k_{x,L} - 2\pi/a_0$. Here, $k_{x,L}$ denotes the wavevector component of the incident far-field light. The associated field vectors mostly point in z-direction. In addition to these delocalized fields, highly localized field enhancements are seen at the edges of the slits, as shown in detail in Fig. S9 (left).

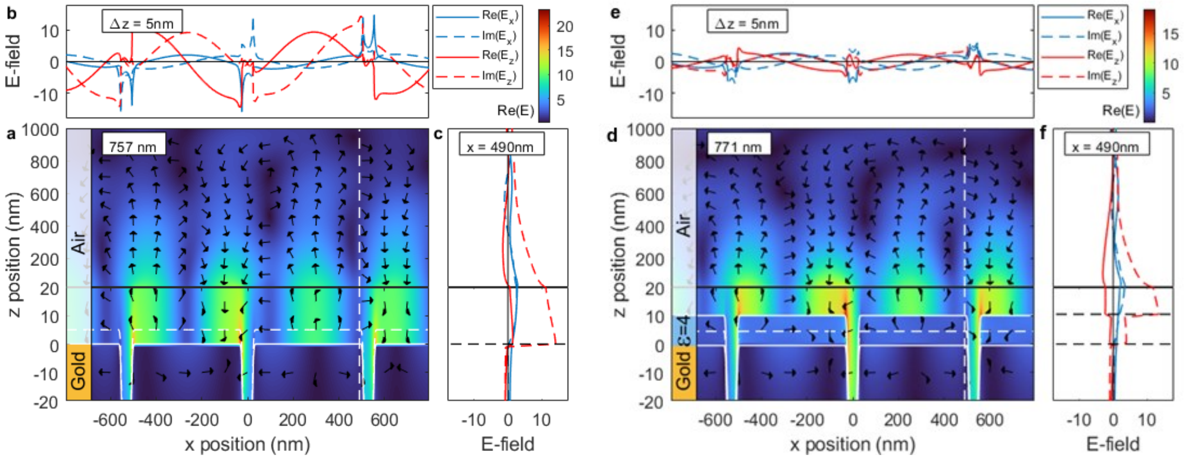

Supplementary Figure 8: *Same as in Fig. S6 but with a structured gold film containing an array of nanoslits with 530 nm period. The simulations are performed for gratings without any coating in panel a), b) and c) and with a 10-nm-thick dielectric coating*

with $\varepsilon_{Diel} = 4$ in panel d), e) and f). The cross sections in b) and e) are taken at a constant height of $\Delta z = 5$ nm above the gold surface. The spatial field distributions are shown at the wavelength of the AM[-1] SPP resonance with a field enhancement of ~10. In addition, field enhancements are seen near the edges of the slits. Inside the dielectric coating, the z-component of the field is strongly reduced.

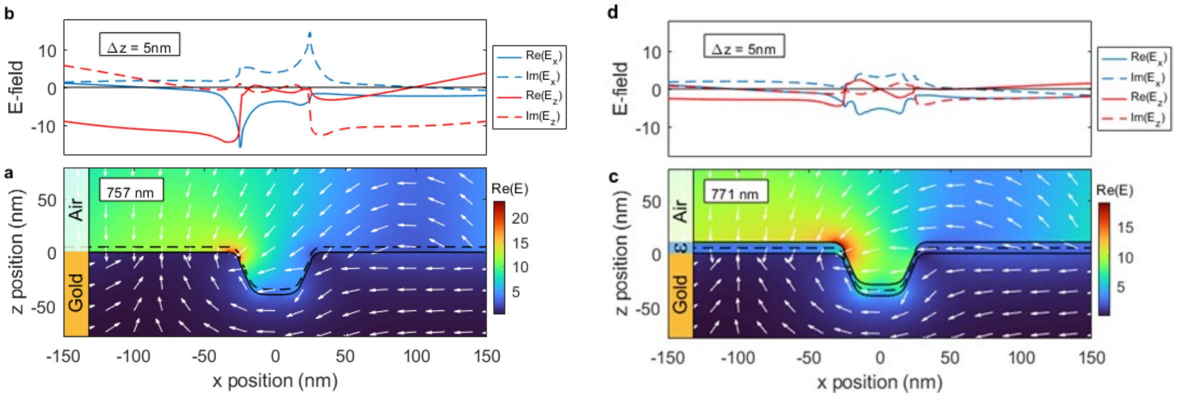

Supplementary Figure 9: *Close up view of the local electric field near the central groove. a) Real-value of the electric field at 757 nm. b) Crosscuts a height 5 nm above the gold film. c) Real value of the electric field at 771 nm. d) Crosscuts at a height of 5 nm above the gold film. The fields are taken from Fig. S8 for the case of the bare gold grating in panel a) and b) and that covered with a 10-nm-thick dielectric layer in panel c) and d).*

To analyze the effect of the background dielectric function of the 10-nm-thick J-aggregated squaraine thin film on the field distributions, we performed simulations for a nanoslit array coated with a 10-nm thick dielectric with $\varepsilon_{Diel} = 4$. Since the coating slightly shifts the SPP resonance, the fields are analyzed at a wavelength of 771 nm. This corresponds to the resonant excitation of the AM[-1] mode, now slightly detuned in energy due to the presence of the thin dielectric coating. Figs. S8 (right) and S9 (right) show the field distributions for the coated array. The field components normal to the surface are largely reduced inside the dielectric coating.

Fig. S10 presents the effect of the dielectric environment on the x- and z-components of the field that has been obtained by spatial integration over a height of 10 nm above the gold film. For regions outside of the slits, the z-component is reduced by a factor of 4, whereas the x-component remains essentially unchanged.

In both cases, we see that the x-component of the field is locally enhanced inside the slit region by roughly a factor of three. We argue below that the enhanced E_x component inside the slits makes the most significant contribution to the strong exciton-plasmon coupling in our arrays.

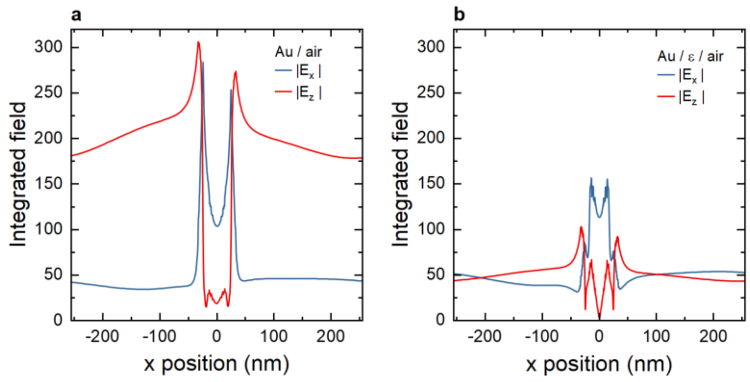

Supplementary Figure 10: *Magnitudes of the x- (blue line) and z- (red line) components of the electric field on top of the gold grating, spatially integrated over the first 10 nm above the surface of the gold nanoslit array, shown for a) an uncoated grating (Fig. S8 left) and b) for a grating with dielectric coating (Fig. S8 right). a) For the uncoated array, the z-component dominates, especially in the regions outside of the slits. b) For a 10-nm dielectric coating, both the x- and z-components are reduced. The dielectric layer mostly screens the z-component, strongly reducing its strength relative to that of the x-component.*

7.3. Anisotropic exciton-plasmon coupling

We first show that the z-component of the electric field makes a vanishingly small contribution to all optical signals seen in our work. For this, we performed a simulation of the angle-resolved linear reflectivity of a nanoslit array covered with a fictitious, anisotropic and uniaxial J-aggregate layer of 10-nm thickness. Its optical axis is assumed to be oriented along the z axis and its dielectric tensor is taken as $\overleftrightarrow{\varepsilon} = \overleftrightarrow{I} + \chi_{Jagg}(\omega)\, \hat{z}\hat{z}$ (Fig. S11a). In this case, the far field light couples only to the SPP mode. The SPP dispersion is similar to that in Fig. S1b, except for a small energetic shift. Importantly, it shows basically no sign of a coupling to the J-aggregate excitons. Also, the direct coupling between far field light and excitons is so weak that the "uncoupled" exciton peak in Fig. 1 is absent in Fig. S11a. Hence, the z-component of the electric field can safely be neglected when discussing exciton-plasmon coupling effects.

For comparison, the optical axis of the J-aggregate layer is now assumed to be oriented along the x-axis and to have a dielectric tensor of the form $\overleftrightarrow{\varepsilon} = \overleftrightarrow{I} + \chi_{Jagg}(\omega)\, \hat{x}\hat{x}$ (Fig. S11b). In this case, strong coupling and polariton formation appears with a normal mode splitting virtually identical to that in Fig. 1. Also, the angle-independent signature of the "uncoupled excitons" is clearly visible. Evidently, the local in-plane, x-components of the electric field vector govern the strong coupling between excitons and SPPs.

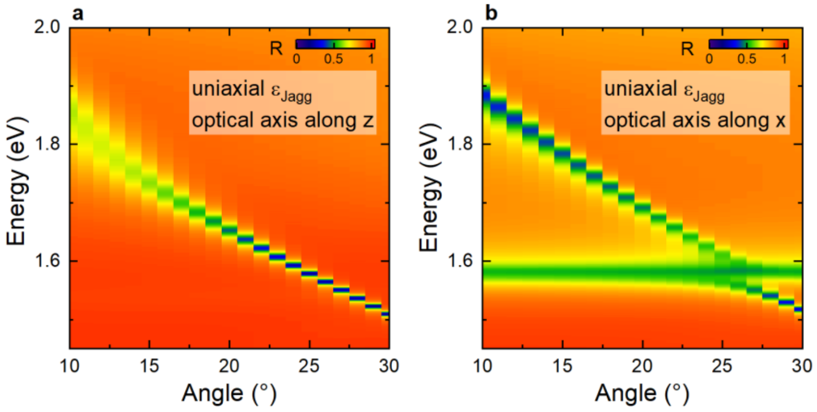

***Supplementary Figure 11**: a) Simulated angle-dependent reflectivity spectra of a gold nanoslit array (parameters as in Fig. S1), covered with a uniaxial thin film with optical axis oriented along z. While the dip in reflectivity due to the coupling to the grating SPPs is strong, coupling to the J-aggregate exciton resonance is virtually absent. b) The same spectra calculated for a uniaxial layer with optical axis oriented along x. Now, formation of upper and lower polaritons and coupling to uncoupled excitons of the thin film appears.*

7.4. Strongly and weakly coupled excitons

In the final the step, we try to distinguish between the role of excitons inside the slits and excitons that are placed between the slits. For this, we perform FDTD simulations with an isotropic J-aggregate film with dielectric function $\varepsilon_{\text{Jagg}}(\omega)$. We deposit this film either inside the slits, in a 120-nm-wide region (Fig. S12a), or in the 410-nm wide region between neighboring slits (Fig. S12b). The 10-nm thickness of the layer is the same as in all other simulations. In the case that the J-aggregates are only placed inside the slits, the entire oscillator strength is transferred to the polaritons while the states near the X energy are optically dark.

Strong coupling and polariton formation are clearly observed. The normal mode splitting is, however, somewhat smaller than in the simulations shown in Fig. 1. As in experiment, the LP transition is optically bright and increases in amplitude beyond the crossing angle. In contrast, the UP peak vanishes almost completely for angles larger than 25°. Again, this agrees well with the experimental observation.

This scenario changes quite substantially when placing the J-aggregate excitons in the region between two slits (Fig. S12b). Now, the “uncoupled” exciton peak is clearly visible. This implies that, now some of the states near the X energy have a finite oscillator strength. The spectra also show a weak anticrossing between UP and LP.

Now, however, the UP peak is bright beyond the crossing while the LP peak is much reduced in amplitude. This is in stark contrast to the experimentally measured angle-resolved linear reflectivity. The flip in brightness between UP and LP, most obvious at angles beyond the crossing, points to a change of sign of the coupling matrix element between J-aggregate excitons and plasmons [18]. This sign change may reflect the finite phase that is accumulated by the SPP wave upon propagating from the slit, launching the SPP, to the J-aggregate emitter and then to the nearest neighbor slit which scatters the SPP into the far field.

In summary, this suggests that J-aggregate excitons inside the slit region predominantly contribute to polariton formation by coupling to the in-plane component of the local optical field. In contrast, the uncoupled X peak that is seen in the linear dispersion arises excitons in between the slits with finite oscillator strength.

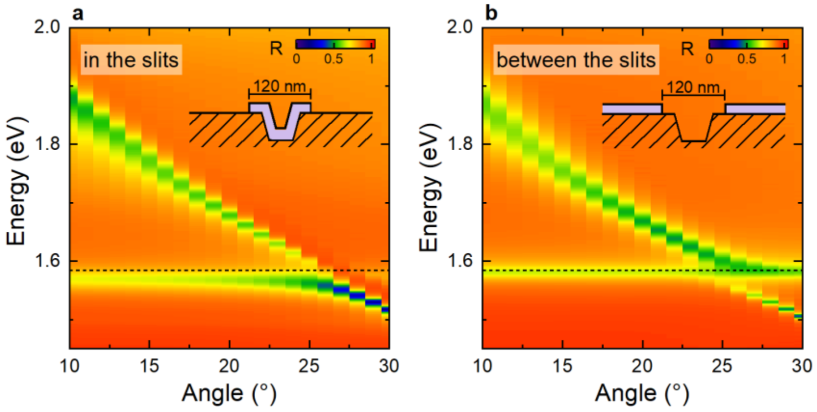

__Supplementary Figure 12__: Simulated angle-dependent reflectivity spectra of a gold nanoslit array (parameter as in Fig. S1), covered with an isotropic film with 10-nm thickness and dielectric function $\varepsilon_{Jagg}(\omega)$. a) Only the slit region of the grating with a width of 120 nm is covered with the film. The spectra reveal polariton formation with a normal mode splitting that is slightly smaller than in Fig. 1. "Uncoupled" exciton peaks are absent. For incident angles beyond the crossing angle, the LP resonance is optically bright while the UP has negligible oscillator strength. b) The same spectra calculated for a film deposited in the region between neighboring slits. In addition to a weak polariton splitting close to the crossing angle, the spectra also show an angle-independent "uncoupled exciton" peak. Now, the LP amplitude vanishes at angles below the crossing while the UP gains oscillator strength beyond the crossing.

Supplementary Note 8: Frenkel exciton simulations

To model the coupling of the molecular J-aggregate with the plasmon mode we perform qualitative microscopic simulations based on a disordered Frenkel exciton model. We choose $N = 300$ squaraine monomer states at $E_{SQ} = 1.923$ eV with Gaussian disorder in site energy of $\sigma = 15.4$ meV [19]. For simplicity, we set the monomer transition dipole moment to $\mu_{SQ} = 1/\sqrt{N}$.

The plasmon mode is introduced as a single state $|P\rangle$ with energy E_P and transition dipole moment μ_P. The Frenkel exciton Hamiltonian then reads

$$\hat{H}_F = E_P|P\rangle\langle P| + \sum_{n=1}^{N}\big(E_n|n\rangle\langle n| + V_{XP,n}(|n\rangle\langle P| + |P\rangle\langle n|)\big) + \sum_{n,m=1}^{N} J_{nm}|n\rangle\langle m|, \qquad (8)$$

where $V_{XP,n} = -\boldsymbol{\mu}_{\mathbf{SQ}} \cdot \mathbf{E}_{\mathbf{SPP,n}}$ denotes a local exciton plasmon coupling between the monomer state $|n\rangle$ at site *n* and the plasmon field $\mathbf{E}_{\mathbf{SPP,n}}$ at the same site. We choose a dipolar coupling between monomer states of $J = -166$ meV, and limit this coupling to nearest neighbors by setting $J_{n,m} = J\delta_{n,m\pm1}$. Periodic boundary conditions are applied. For $V_{XP,n} = 0$, Eq. (S8) thus describes a superradiant J-aggregate exciton state with $E_X = 1.59$ eV determined by the nearest-neighbor coupling between the monomers, as shown in Fig. S13b [5]. Disorder in site energy results in Anderson localized segments of around 24 monomers within the linear chain [6] (Fig. S13a).

To study the effect of a spatially structured plasmon field on the optical properties and time dynamics of the coupled system, we choose the local X-P couplings $V_{XP,n}$ to be proportional to the amplitude of the plasmon field at each monomer site n. In agreement with the results of the FDTD simulations, as discussed above (section 7), we assume that the local coupling is governed by the in-plane components of the local SPP field. The FDTD simulations in Fig. S12 suggest that the plasmon nearfield is enhanced in the slit region compared to the region between the slits. As such, we divide the X-P coupling into two regions: $V_{S,n}$ for the strong coupling inside the slits and $V_{W,n}$ for a weaker coupling in the region in-between two slits. In

the following simulations, $V_{S,n}$ and $V_{W,n}$ are taken as constant and real-valued parameters. The number of monomers in the two regions is of N_S and N_W, respectively.

8.1. Reduced Hamiltonian

For rationalizing the results of this Frenkel exciton model, we introduce an effective Hamiltonian

$$\hat{H}_{red} = E_P|P\rangle\langle P| + E_{X_S}|X_S\rangle\langle X_S| + E_{X_W}|X_W\rangle\langle X_W| + V_S(|X_S\rangle\langle P| + |P\rangle\langle X_S|) + V_W(|X_W\rangle\langle P| + |P\rangle\langle X_W|) \tag{9}$$

comprising four states $|0\rangle$, $|P\rangle$, $|X_S\rangle$ and $|X_W\rangle$, where $|X_S\rangle$ and $|X_W\rangle$ indicate strongly coupled (inside the slits) and weakly coupled excitons (in between two slits), respectively. The ground state energy of the system is set to zero. The exciton energies are approximated from the Frenkel exciton parameters as $E_{X_S} = E_{X_W} = E_{SQ} - 2J$. The collective transition dipole moments and coupling strengths can be calculated using the number of monomers per area and their individual transition dipole moments and X-P coupling strengths. We thus obtain $\mu_{X_S} = \sqrt{N_s}\mu_{SQ}$ and $\mu_{X_W} = \sqrt{N_W}\mu_{SQ}$ for the transition dipole moments, and $V_S = \sqrt{N_S}V_{S,n}$ and $V_W = \sqrt{N_W}V_{W,n}$ for the coupling strengths.

8.2. Simulating the optical spectra and dynamics upon optical excitation

We simulate the dynamics of both the full Frenkel and the reduced effective Hamiltonian following optical excitation by numerically integrating the master equation in Lindblad form [5, 16]

$$\dot{\hat{\rho}} = -\frac{i}{\hbar}[\hat{H}, \hat{\rho}] + \frac{1}{2}\sum_k \left(2\hat{L}_k\hat{\rho}\hat{L}_k^\dagger - \hat{L}_k^\dagger\hat{L}_k\hat{\rho} - \hat{\rho}\hat{L}_k^\dagger\hat{L}_k\right). \tag{10}$$

In Eq. (S10), the full Hamiltonian $\hat{H} = \hat{H}_S + \hat{H}_{int}(t)$ describes the free evolution of the system via $\hat{H}_S$, which can be either $\hat{H}_F$ or $\hat{H}_{red}$, and its light-matter interaction. The latter is governed by the time-dependent interaction Hamiltonian

$$\hat{H}_{int}(t) = -\hat{\mu}E(t) \tag{11}$$

which accounts for optical excitation of the system by an external light field in dipole approximation. In Eq. (S11), $\hat{\mu}$ denotes the transition dipole moment operator of the system. We assume a short and sufficiently weak laser pulse at 1.6 eV with a pulse duration of 5 fs (FWHM of the intensity profile). Dephasing and relaxation processes are incorporated through appropriate Lindblad operators $\hat{L}_k$ [20, 21]. In the following we consider electronic dephasing with $\hat{L}_d = \sqrt{2\gamma}\sum_n|n\rangle\langle n|$.

This dephasing adds a term $-\gamma\sum_{n=1}^{N}(|0\rangle\langle n| + |n\rangle\langle 0|)$ to Eq. (S10), which exponentially dampens coherences of the density matrix between the ground state and all excited states while maintaining coherences within the excited state manifold. In the simulations, we set $\gamma = 8\ \mathrm{meV}$. Numerical integration of Eq. (S10) yields the full time-dependence of $\hat{\rho}$. Optical absorption spectra are calculated from this density matrix via the expectation value of the transition dipole moment operator, which corresponds to the time-domain polarization

$$P(t) = Tr(\hat{\mu}\hat{\rho}(t)). \tag{12}$$

Since the optical excitation pulses are much shorter in time than the system response, we approximate the Fourier transform of this time-domain signal as the susceptibility $\chi(\omega)$. Its imaginary part thus yields the optical absorption spectrum.

8.3. Simulation parameters

The simulation results based on the full Frenkel exciton and the reduced effective Hamiltonian are depicted in Fig. 4 of the main manuscript. For these simulations, we have chosen a plasmon energy of $E_P = 1.627\ \mathrm{eV}$, corresponding to an incidence angle of 21°. We have approximated the X-P coupling as $V_{S,n} = 5\ \mathrm{meV}$ and $V_{W,n} = 0.5\ \mathrm{meV}$ with $N_S = 30$ and $N_W = 270$ strongly and weakly coupled monomer states, respectively. The plasmon transition dipole moment was set to $\mu_P = 0.7$, smaller than the combined dipole moment of the $N = 300$ squaraine monomers of $\sqrt{N}\mu_{SQ} = 1$.

These parameters give rise to effective parameters of $V_S = -27.4\ \mathrm{meV}$, $V_W = -8.2\ \mathrm{meV}$, $\mu_S = 0.32$, $\mu_W = 0.95$ and $\mu_P = 0.7$. The comparison of both the optical properties and the time-dependent population dynamics of the full Frenkel exciton simulation and the effective system in Fig. 4 shows a good agreement between the two models. This justifies the use of the reduced system for further simulations of the nonlinear signals.

To show the effect of the X-P coupling on the exciton dynamics, we report an additional simulation without plasmon state in Fig. S13. In this case, we see the typical disorder induced localization of J-aggregated excitons. In this case, of course, there is no difference in the localization behaviour inside and outside of the slit region (dashed lines). Partial quantum beats in the local exciton population result from a finite spatial overlap of neighboring localized exciton wavefunctions. No correlations between quantum beats and Rabi oscillation period exist. Now, the optical absorption spectrum (Fig. S13b) shows only the weakly inhomogeneously broadened J-aggregated exciton peak.

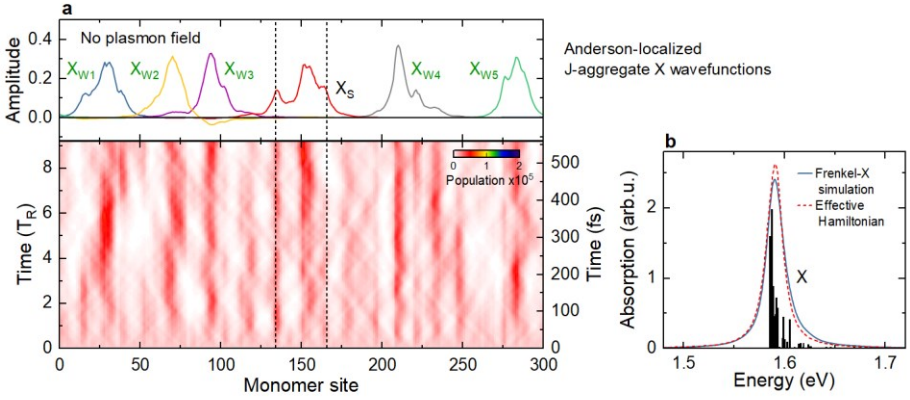

***Supplementary Figure 13**: Frenkel exciton simulations without plasmon using the same simulation parameters as in Fig. 4. a) Wavefunctions of the six lowest energy X states (upper panel). The Anderson-localized excitons X_{W1}-X_{W5} are localized outside of the slit region, while the exciton X_S is mainly localized inside the slit region (dashed lines). The spatio-temporal population dynamics (bottom panel) after impulsive excitation also show partial quantum beats resulting from a finite spatial overlap of neighboring exciton wavefunctions. These dynamics are distinctly different from the Rabi oscillations dynamics observed in the presence of plasmon coupling. b) Simulated absorption spectrum resulting from the Frenkel exciton simulation (blue) and the reduced effective Hamiltonian (red). The oscillator strengths of the individual transitions are plotted as black bars.*

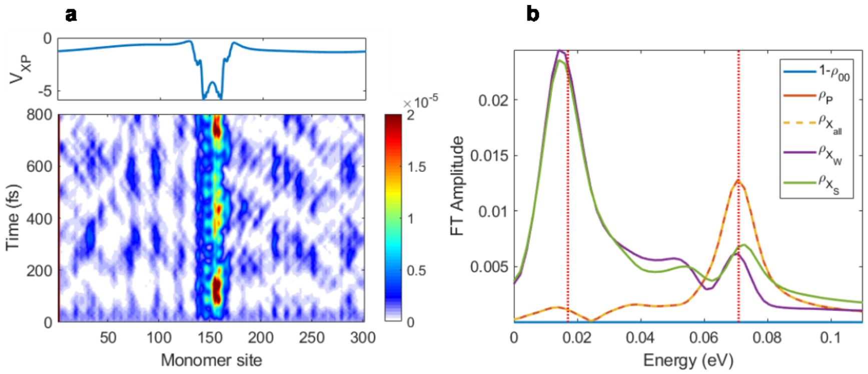

***Supplementary Figure 14**: Frenkel exciton simulations of the local exciton density considering a spatially inhomogeneous field distribution in the region outside the slits. This distribution mimics more closely the SPP field distribution in Fig. S10. a) Again, we see fast Rabi oscillations in the slits, superimposed by slow oscillations between regions outside and inside the slits. b) The Fourier transforms of the population oscillations clearly show the same two distinct classes of Rabi oscillations as in Fig. 4.*

Supplementary Note 9: Simulation of 2DES and pump-probe spectra

To simulate the experimental nonlinear signals, in particular the pump-probe and 2DES maps, we compute the full dynamics of the density matrix $\hat{\rho}$ after a series of interactions with up to three laser pulses. From this, we then calculate the sample polarization and the resulting optical spectra [22].

For the numerical integration of the master equation in Lindblad form, Eq. (S10), we use a non-perturbative approach [3, 21]. The total electric field, entering the interaction Hamiltonian in Eq. (S11),

$$E(t) = \sum_{\substack{n=pu1,\\pu2,pr}} E_{0,n} \mathrm{e}^{-2\ln 2\left(\frac{t-t'_n}{\Delta t}\right)^2} \cos(\omega_L(t-t'_n)+\phi_n) \tag{13}$$

comprises of up to three laser pulses (pump 1, pump 2 and probe) with amplitude $E_{0,n}$, pulse duration $\Delta t = 10$ fs, frequency $\omega_L = 1.6\,\mathrm{eV}/\hbar$, phase ϕ_n for phase cycling and a time shift t'_n. Following the experimental data acquisition scheme (section 4), we label the delay between the pump pulses as the coherence time $\tau = t'_{pu2} - t'_{pu1}$ and the delay between the second pump pulse and the probe pulse as the waiting time $T = t'_{pr} - t'_{pu2}$. A detection time t of zero corresponds to the arrival time of the probe pulse. The detection time axis coincides with the one used for the numerical integration of Eq. (S10).

In the experiment, the collinear pump pulses with wavevector $\mathbf{k_{pu}}$ impinging on the sample under a small angle with respect to the probe pulse with $\mathbf{k_{pr}}$. We collect all emitted polarizations in the direction $\mathbf{k'_{pr}}$ of the reflected probe. By chopping the pump pulses, we can either record a linear optical spectrum or a nonlinear one. In the simulations, we calculate the linear polarization $P^{(1)}(t)$ without pump, or a total polarization $P^{tot}(\tau, T, t)$ following Eq. (12), including all nonlinear signals that arise from all possible interactions with the three pulses [22]. We then deduce linear and total susceptibilities as a function of the detection energy E_{det} from these polarizations via a Fourier transform along the detection time t

$$\chi^{(1)}(E_{det}) = \frac{1}{\varepsilon_0}\mathcal{F}\big(P^{(1)}(t)\big)/\mathcal{F}\big(E_{pr}(t)\big) \tag{14}$$

$$\chi^{tot}(\tau, T, E_{det}) = \frac{1}{\varepsilon_0}\mathcal{F}(P^{tot}(\tau, T, t))/\mathcal{F}\big(E_{pr}(t)\big) \tag{15}$$

where ε_0 denotes the vacuum dielectric constant.

While we rely on directional phase matching in the experimental setup, we employ a phase-cycling scheme in our simulations to isolate the desired linear and nonlinear contributions to our signal. For this, we calculate $P^{tot}(\tau, T, t)$ for four different phase settings of $\phi_{pu1} = \phi_{pu2} = \left[0, \frac{\pi}{2}, \pi, \frac{3\pi}{2}\right]$ for both pump pulses, with $\phi_{pr} = 0$ [3, 23, 24]. We then perform the average over these four settings to obtain the phase-cycled average $\chi_{PC}^{tot}(\tau, T, E_{det})$. This numerical phase-cycling scheme allows for extracting the contributions to the nonlinear signal corresponding to those that are measured in the partially collinear experimental geometry. After applying this phase-cycling scheme and after subtracting the linear contribution, we obtain the nonlinear signal as

$$\chi^{nl}(\tau, T, E_{det}) = \chi_{PC}^{tot}(\tau, T, E_{det}) - \chi^{(1)}(E_{det}). \tag{16}$$

From this nonlinear susceptibility, we calculate the 2DES map as a function of the coherence time as

$$S_{2D}(\tau, T, E_{det}) = \Im\big(\chi^{nl}(\tau, T, E_{det})\big). \tag{17}$$

Finally, energy-energy 2DES maps for a fixed waiting time T are obtained by taking the real part of the Fourier transform along the coherence time τ

$$S_{2D}(E_{ex}, T, E_{det}) = \Re\big(\int_{-\infty}^{\infty} \theta(\tau) S_{2D}(\tau, T, E_{det}) \mathrm{e}^{iE_{ex}\tau/\hbar} \mathrm{d}\tau\big), \tag{18}$$

yielding the excitation energy axis E_{ex}. Setting $\tau = 0$ in the simulations allows for calculating pump-probe spectra as a function of E_{det} and T.

9.1. Plasmon

While the inclusion of one-quantum states, as used in Eqs. (S8) and (S9), is sufficient for the discussion of the linear optical spectra, higher order quantum excitations are necessary for accurately describing the nonlinear spectra. For this, we express the SPP Hamiltonian in second quantization as a harmonic oscillator (HO) [25]

$$\hat{H}_P = E_P(\theta) \hat{b}_P^\dagger \hat{b}_P \tag{19}$$

with plasmon creation and annihilation operators $\hat{b}_P^\dagger$ and $\hat{b}_P$, respectively. Angle-dependent plasmon energies $E_P(\theta)$ are deduced from the linear reflectivity measurements of the gold nanoslit array with and without J-aggregate coating.

We describe the coupling of far field light to the SPP by introducing a transition dipole moment μ_P to quantify the coupling strength. The dipole moment is estimated from the FDTD simulations to be ~500-800 Debye, depending on the angle of incidence. We write a plasmon dipole moment operator as

$$\hat{\mu}_P = \mu_P\big(\hat{b}_P^\dagger + \hat{b}_P\big). \tag{20}$$

The radiative SPP lifetime is accounted for via the Lindblad relaxation term

$$\hat{L}_{rel,P} = \sqrt{\kappa_P} \hat{b}_P, \tag{21}$$

which introduces plasmon population relaxation and thus results in a finite linewidth of the optical spectra. We neglect pure dephasing of the plasmon transitions [26]. We also disregard possible inhomogeneous broadening effects that may be caused by manufacturing-related imperfections and finite angular spread of the weakly focused laser beam in the experiment. We choose a lifetime

$$T_{1,P} = 1/\kappa_P \tag{22}$$

of 150 fs to best match the experimentally observed decay dynamics. This slightly underestimates the plasmon linewidth in the optical spectra.

9.2. Excitons

For conceptual simplicity, we introduce two distinct classes of J-aggregate exciton manifolds, termed $X = \{X_S, X_W\}$. We consider X_S as representative for those excitons that reside inside the slits and couple strongly to the local SPP field. In contrast, we use X_W for weakly-coupled excitons between the slit. We estimate that each of these aggregated excitons is delocalized, in the absence of the SPP field, over ~24 monomer units [11]. In agreement with the current theoretical understanding of J-aggregates [5, 6] and our experimental observations [27], we assume that the energy of the corresponding two-exciton (XX) states is slightly larger than twice the exciton energy. This two-exciton blue-shift arises from Pauli blocking within the chain of fermionic monomers [5]. This Pauli-blocking and the resulting XX blue-shift introduces an optical nonlinearity into the system. We can model these states using bosonic HO operators for each of the excitons and account for fermionic manybody effects using an excitonic interaction Hamiltonian $\widehat{H}_{XX} = \Delta E|XX\rangle\langle XX|$. This interaction Hamiltonian introduces the energy correction of the XX states due to the Pauli blocking effects [6, 28]. Such manybody interactions transform the exciton Hamiltonian into an anharmonic one

$$\widehat{H}_X = E_X \hat{b}_X^\dagger \hat{b}_X + \widehat{H}_{XX}. \tag{23}$$

Since our pump-probe and 2DES measurements are performed in the regime of third-order perturbation theory, we can neglect higher-lying exciton states beyond the XX states.

The exciton energy is set to $E_X = 1.587$ eV to match with experiment. We estimate the blueshift between the X and XX states to be around $\Delta\mathrm{E} = 5\ \mathrm{meV}$ [29], giving a two-exciton energy of $E_{XX} = 2E_X + \Delta E$. Radiative lifetime and pure dephasing of the excitons are taken into account using the respective Lindblad operators

$$\hat{L}_{rel,X} = \sqrt{\kappa_X}\hat{b}_X \tag{24}$$

$$\hat{L}_{dep,X} = \sqrt{2\gamma_X}\hat{b}_X^\dagger \hat{b}_X. \tag{25}$$

A pump-probe map (Fig. S15b) of a bare X system that is simulated using these parameters shows good agreement with experiment (Fig. S5a). Both the energy and the spectral linewidth of the main dispersive resonance match well. This dispersive peak results from the superposition of both, ground-state bleaching (GSB) and stimulated emission (SE) of the one-exciton (0 → X) transition, and the blue-shifted excited-state absorption (ESA) of the two-exciton (X → XX) transition. Also, its characteristic perturbed free induction decay (PFID) [5] at negative waiting times is well reproduced when choosing a pure dephasing time

$$T_{2,X}^* = 1/\gamma_X \tag{26}$$

of 80 fs. In these simulations, we have neglected inhomogeneous broadening effects and set the radiative lifetime of the excitons to $T_{1,X} = 10\ ps$.

We write the exciton transition dipole moment operator as

$$\hat{\mu}_X = \mu_X(\hat{b}_X^\dagger + \hat{b}_X), \tag{27}$$

where μ_X is the dipole moment of the $0 \rightarrow \mathrm{X}$ transition. The resulting dipole moment for the $\mathrm{X} \rightarrow \mathrm{XX}$ transition is thus enhanced to $\sqrt{2}\mu_X$. Thus, the exciton behaves as a purely linear harmonic oscillator in the limit of a two-exciton shift $\Delta E \rightarrow 0$. In this case, the energetically overlapping SE/GSB and ESA signals cancel and the nonlinearity vanishes, exactly as it is the case for the harmonic SPP oscillator. A finite blueshift of the XX state now introduces a nonlinearity in the form of a dispersive lineshape (Fig. S15c). The ratio between GSB+SE and ESA signals obtained from Eq. (S27) is the same as derived in the Frenkel exciton description when incorporating two-quantum states and neglecting disorder [5, 6, 16]. Using this model, we see a good agreement between the experimental (Fig. 1c) and simulated (Fig. S15a) 2DES maps for the bare J-aggregated exciton system.

To test that the nonlinearity vanishes for a purely plasmonic system, we performed simulations for a SPP HO and compared them to that of the "anharmonic" exciton oscillator. Fig. S15c (red line) compares the simulated plasmon nonlinearity, enhanced by a scaling factor of 1000, to that of the exciton under the same excitation conditions and using identical transition dipole moments (500 D) and energies. In the simulations, the electric fields of the excitation pulses are chosen sufficiently weak to deplete the ground state by less than $2 \cdot 10^{-5}$. The finite residual signal arises from the truncation of the Hamiltonian beyond the 2-quantum manifold. It is so weak that it has a negligible effect on the simulated pump-probe and 2DES spectra. This allows us to ignore 3-quantum and higher-lying states in our simulations.

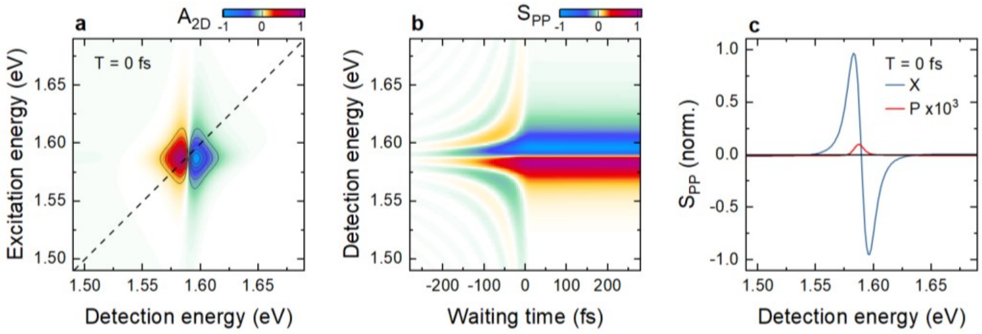

Supplementary Figure 15: *Simulated nonlinear signals of a bare squaraine J-aggregate exciton (X) system, compared to that of a plasmonic nanoslit array (P). A transition dipole moment of 500 D was chosen for both the X and the P system. The ground state depletion after optical excitation amounts to* $<2 \cdot 10^{-5}$ *in both cases. a) 2DES map at T = 0 fs for the J-aggregate exciton, showing a dispersive line along the detection energy due to the blueshift of the XX state by* $\Delta E = 5\ meV$*. b) Pump-probe simulation showing the characteristic dispersive exciton lineshape for positive waiting times. The pronounced oscillatory features at negative time reflect the PFID of the X transition. c) Crosscut of the pump-probe map in b) at T=0 fs (blue) and of that of a nanoslit array P (red). Small residual plasmon nonlinearities arise from the truncation of the Hamiltonian beyond the two-quantum manifold.*

9.3. Exciton-plasmon coupling

Dipolar interactions of the strongly and weakly coupled excitons with the plasmon field are introduced by adding two coupling Hamiltonians in rotating wave approximation, following [20]

$$\hat{H}_{X_W P} = V_W\left(\hat{b}_P^\dagger \hat{b}_{X_W} + \hat{b}_{X_W}^\dagger \hat{b}_P\right) \tag{28}$$

$$\hat{H}_{X_S P} = V_S\left(\hat{b}_P^\dagger \hat{b}_{X_S} + \hat{b}_{X_S}^\dagger \hat{b}_P\right). \tag{29}$$

Coupling between the two excitons is neglected. We also add phenomenological cross-damping terms to account for super- and subradiance phenomena [28]

$$\dot{\hat{\rho}}_C = \frac{1}{2}\kappa_{XP}\left(2\hat{b}_P\hat{\rho}\hat{b}_X^\dagger - \hat{b}_X^\dagger\hat{b}_P\hat{\rho} - \hat{\rho}\hat{b}_X^\dagger\hat{b}_P\right) + \frac{1}{2}\kappa_{XP}\left(2\hat{b}_X\hat{\rho}\hat{b}_P^\dagger - \hat{b}_P^\dagger\hat{b}_X\hat{\rho} - \hat{\rho}\hat{b}_P^\dagger\hat{b}_X\right) \tag{30}$$

with $\kappa_{XP} = \sqrt{\kappa_X\kappa_P}$, for both the weakly and strongly coupled excitons. Since we assume an exciton lifetime of 10 ps, these cross-damping terms have a negligible effect on the simulated spectra.

9.4. Total Hamiltonian

The total system Hamiltonian used for the pump-probe and 2DES simulations then finally reads

$$\hat{H}_S = \hat{H}_P + \hat{H}_{X_S} + \hat{H}_{X_W} + \hat{H}_{X_SP} + \hat{H}_{X_WP}. \tag{31}$$

The interaction Hamiltonian follows Eq. (S11) and reads as

$$\hat{H}_{int} = -\left(\hat{\mu}_P + \hat{\mu}_{X_S} + \hat{\mu}_{X_W}\right)E(t). \tag{32}$$

All simulations are performed in a three-particle basis comprising the states $|n_P, n_{X_S}, n_{X_W}\rangle$ with $n_P = \{0, P, 2P, \dots\}$, $n_{X_S} = \{0, X_S, XX_S, \dots\}$ and $n_{X_W} = \{0, X_W, XX_W, \dots\}$. We can sort these states by the number of total excitations of the system, giving one ground state, $|0,0,0\rangle$, three one-quantum (1Q) states ($|P,0,0\rangle$, $|0,X_S,0\rangle$ and $|0,0,X_W\rangle$) and six two-quantum (2Q) states ($|2P,0,0\rangle$, $|P,X_S,0\rangle$, $|P,0,X_W\rangle$, $|0,XX_S,0\rangle$, $|0,X_S,X_W\rangle$ and $|0,0,XX_W\rangle$).

This Hamiltonian is graphically visualized in the basis of coupled J-aggregate exciton and plasmon states in Fig. S16a. Here, the three 1Q states are taken at the same energy $E_P = E_X$ and the couplings between these states are depicted as green arrows. For the six resulting 2Q states, the two-exciton shifts are indicated schematically. Couplings between the 2Q states occur either with the 1Q couplings V_W and V_S (green arrows) or with couplings that are enhanced by a factor $\sqrt{2}$ (red arrows). Optical transition matrix elements between ground state, 1Q and 2Q states are indicated.

The same Hamiltonian is plotted in a polariton basis in Fig. S16b.

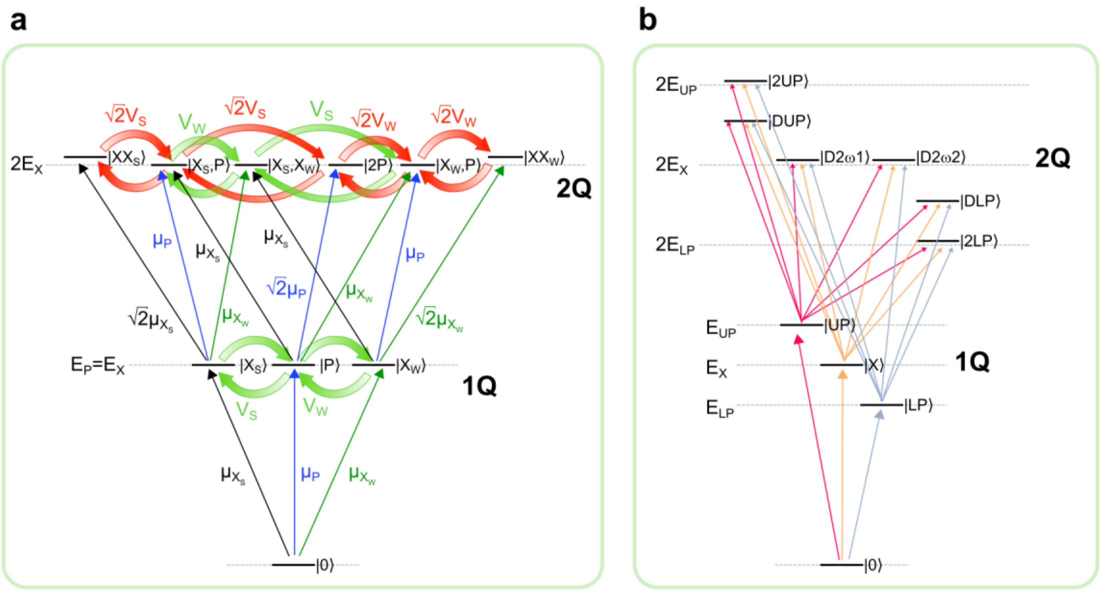

Supplementary Figure 16: *Schematic representation of the full exciton-plasmon Hamiltonian in a) the basis of interacting J-aggregate exciton and plasmon states and b) in the polariton basis.*

9.5. Simulation parameters

To limit the number of free parameters, we fix the plasmon energy at a specific incidence angle θ to that deduced from the experimental linear spectra. The exciton energy ($E_X = 1.587$ eV), two-exciton blueshift ($\Delta E = 5$ meV) and dephasing time ($T^*_{2,X} = 80$ fs) are also fixed. Plasmon and exciton lifetimes are kept constant at $T_{1,P} = 150$ fs and $T_{1,X} = 10$ ps, respectively.

The truncation of the manybody Hamiltonian beyond the two-quantum manifold limits the dimensionality of the Hilbert space to 10. We have carefully checked that the optical excitation conditions of all three pulses are kept sufficiently weak to ensure that higher quantum manifolds do not contribute to the simulated spectra. This is equivalent to restricting the optical response to third order in the external field. In these checks we included the states of three-quantum manifold in the simulations, giving a total of 21 quantum states. Under the chosen excitation conditions, essentially no difference in the simulated nonlinearities can be detected.

From the Frenkel exciton simulations in Section 8, we estimate that a fraction of approximately 10% of all excitons are strongly coupled to the plasmon field. They also indicate that the total oscillator strength of the strongly and weakly coupled excitons exceeds that of the SPP system. This sets important boundaries for the choice of the dipole moments in the simulations. We found that transition dipole moments of $\mu_P = 550$ D for the plasmon and $\mu_{X_W} = 730$ D for the weakly coupled exciton gave reasonable match to experiment for all considered incidence angles. In contrast, it was necessary to vary μ_{X_S}, V_W and V_S slightly with angle. Comparison between simulations and experiments indicated that the coupling constant V_S decreases by roughly a factor of two across the considered range of incidence angles. Table 1 summarizes the parameters that were chosen for reaching qualitative agreement between 2DES and pump-probe experiments and simulations for angles of 18°, 21°, 25° and 27°.

Supplementary Table 1: Parameters that were used for simulated pump-probe and 2DES spectra using the model Hamiltonian defined in the text.

**Angle**	E_P	μ_{X_S}	V_W	V_S
18°	1.682 eV	244 D	14 meV	44 meV
21°	1.619 eV	244 D	10 meV	33 meV
25°	1.557 eV	183 D	7 meV	23.1 meV
27°	1.529 eV	183 D	10 meV	18.5 meV

9.6. Additional simulation results

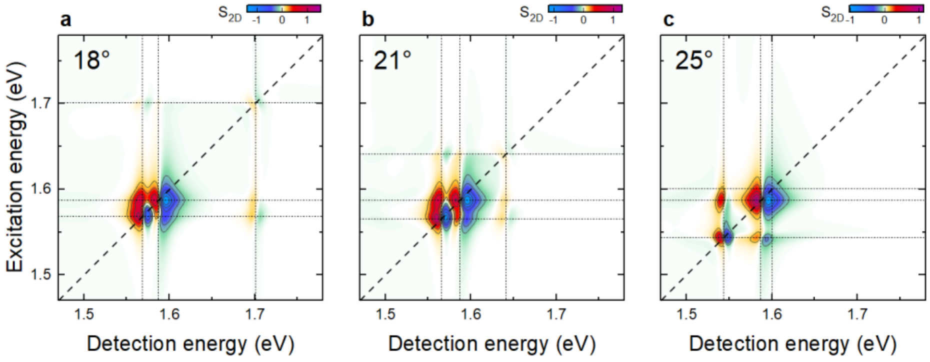

Supplementary Figure 17: *Simulated 2DES maps at T = 0 fs for selected angles of 18° in a), 21° in b) and 25° in c). The dotted lines mark the energies of the lowest three states (LP, X and UP) of the Hamiltonian. Cross-peaks between the LP and X states are more pronounced than those between UP and LP.*

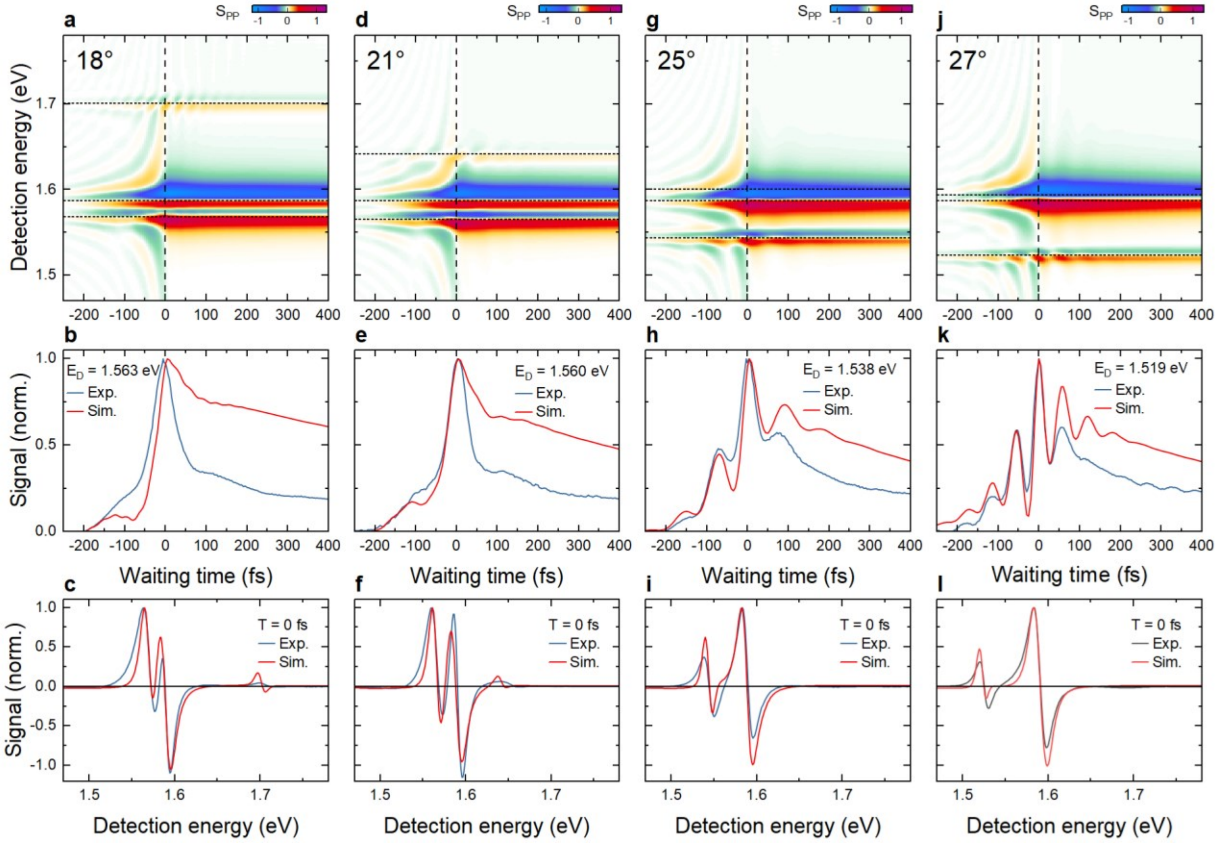

Supplementary Figure 18: *Simulated pump-probe data for strong X-SPP coupling at selected angles. Pump-probe maps (a),d),g),j)) show good agreement with the data in Fig. S5. b),e),h),k) Dynamics of the positive LP peak in the simulations in comparison with experiment. For all angles, the rich oscillatory features are in reasonable agreement at positive and negative waiting times. c),f),i),l) Spectral crosscuts at T = 0 fs for simulated and experimental data showing, with increasing detection energy, dispersive LP, X and UP peaks.*

Supplementary Note 10: UP-LP Rabi oscillations

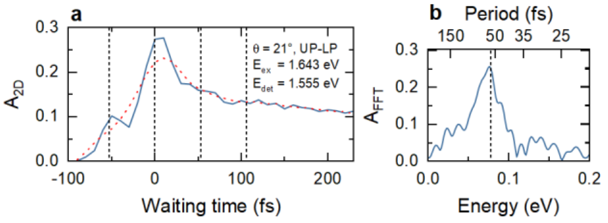

Supplementary Figure 19*: Observation of UP-LP Rabi oscillations at θ = 21°. a) Dynamics of the UP-LP cross-peak in the 2DES measurement. Vertical lines mark oscillations on the temporal trace with 53 fs period, corresponding to an energetic splitting between UP and LP of 78 meV. b) Fourier transform of the residuals in panel a after subtracting the slow background (red dotted line). The dashed line marks the peak energy of 78 meV that corresponds to the UP-LP Rabi oscillation period of 53 fs.*

Supplementary References

1. Schulz, M., Mack, M., Kolloge, O., Lutzen, A. & Schiek, M. Organic photodiodes from homochiral L-proline derived squaraine compounds with strong circular dichroism. *Physical Chemistry Chemical Physics* **19**, 6996-7008 (2017).
2. Schulz, M. *et al.* Giant intrinsic circular dichroism of prolinol-derived squaraine thin films. *Nat. Commun.* **9**, 2413 (2018).
3. Timmer, D. *et al.* Charge Delocalization and Vibronic Couplings in Quadrupolar Squaraine Dyes. *Journal of the American Chemical Society* **144**, 19150-19162 (2022).
4. Terenziani, F., Painelli, A., Katan, C., Charlot, M. & Blanchard-Desce, M. Charge instability in quadrupolar chromophores: Symmetry breaking and solvatochromism. *Journal of the American Chemical Society* **128**, 15742-15755 (2006).
5. Quenzel, T. *et al.* Plasmon-Enhanced Exciton Delocalization in Squaraine-Type Molecular Aggregates. *Acs Nano* **16**, 4693-4704 (2022).
6. Knoester, J. Optical properties of molecular aggregates, in *Organic Nanostructures: Science and Applications* 149-186 (IOS Press, 2002).
7. Zhang, C. *et al.* Nanoaperture fabrication in ultra-smooth single-grain gold films with helium ion beam lithography. *Nanotechnology* **31**, 465302 (2020).
8. Gittinger, M. *et al.* Strongly coupled, high-quality plasmonic dimer antennas fabricated using a sketch-and-peel technique. *Nanophotonics* **9**, 401-412 (2020).
9. Vasa, P. *et al.* Real-time observation of ultrafast Rabi oscillations between excitons and plasmons in metal nanostructures with J-aggregates. *Nat. Photonics* **7**, 128-132 (2013).
10. Ropers, C. *et al.* Femtosecond light transmission and subradiant damping in plasmonic crystals. *Physical Review Letters* **94**, 113901 (2005).
11. Kim, D.S. *et al.* Microscopic origin of surface-plasmon radiation in plasmonic band-gap nanostructures. *Physical Review Letters* **91**, 143901 (2003).
12. Liang, B., Bai, M., Ma, H., Ou, N.M. & Miao, J.G. Wideband Analysis of Periodic Structures at Oblique Incidence by Material Independent FDTD Algorithm. *Ieee T Antenn Propag* **62**, 354-360 (2014).
13. Olmon, R.L. *et al.* Optical dielectric function of gold. *Physical Review B* **86**, 235147 (2012).
14. Grupp, A. *et al.* Broadly tunable ultrafast pump-probe system operating at multi-kHz repetition rate. *Journal of Optics* **20**, 014005 (2018).
15. Brida, D., Manzoni, C. & Cerullo, G. Phase-locked pulses for two-dimensional spectroscopy by a birefringent delay line. *Opt Lett* **37**, 3027-3029 (2012).
16. Hamm, P. & Zanni, M.T. *Concepts and methods of 2d infrared spectroscopy*. (Cambridge University Pres, Cambridge ; New York; 2011).
17. Novotny, L. & Hecht, B. *Principles of nano-optics*. (Cambridge University Press, Cambridge ; New York; 2006).
18. Raether, H. *Surface plasmons on smooth and rough surfaces and on gratings*. (Springer-Verlag, Berlin ; New York; 1988).
19. Kihm, J.E. *et al.* Fabry-Perot tuning of the band-gap polarity in plasmonic crystals. *Physical Review B* **75**, 035414 (2007).
20. Mukamel, S. *Principles of nonlinear optical spectroscopy*. (Oxford University Press, New York; 1995).
21. Palmieri, B., Abramavicius, D. & Mukamel, S. Lindblad equations for strongly coupled populations and coherences in photosynthetic complexes. *Journal of Chemical Physics* **130**, 204512 (2009).
22. Breuer, H.-P. & Petruccione, F. *The theory of open quantum systems*. (Oxford University Press, Oxford ; New York; 2002).

23. Egorova, D., Gelin, M.F. & Domcke, W. Analysis of cross peaks in two-dimensional electronic photon-echo spectroscopy for simple models with vibrations and dissipation. *J. Chem. Phys.* **126**, 074314 (2007).
24. Seidner, L., Stock, G. & Domcke, W. Nonperturbative Approach to Femtosecond Spectroscopy - General-Theory and Application to Multidimensional Nonadiabatic Photoisomerization Processes. *Journal of Chemical Physics* **103**, 3998-4011 (1995).
25. Yan, S.X. & Tan, H.S. Phase cycling schemes for two-dimensional optical spectroscopy with a pump-probe beam geometry. *Chem Phys* **360**, 110-115 (2009).
26. Archambault, A., Marquier, F., Greffet, J.J. & Arnold, C. Quantum theory of spontaneous and stimulated emission of surface plasmons. *Physical Review B* **82**, 035411 (2010).
27. Spano, F.C. & Mukamel, S. Cooperative Nonlinear Optical-Response of Molecular Aggregates - Crossover to Bulk Behavior. *Physical Review Letters* **66**, 1197-1200 (1991).
28. Autry, T.M. *et al.* Excitation Ladder of Cavity Polaritons. *Physical Review Letters* **125**, 067403 (2020).
29. Garraway, B.M. The Dicke model in quantum optics: Dicke model revisited. *Philos T R Soc A* **369**, 1137-1155 (2011).
